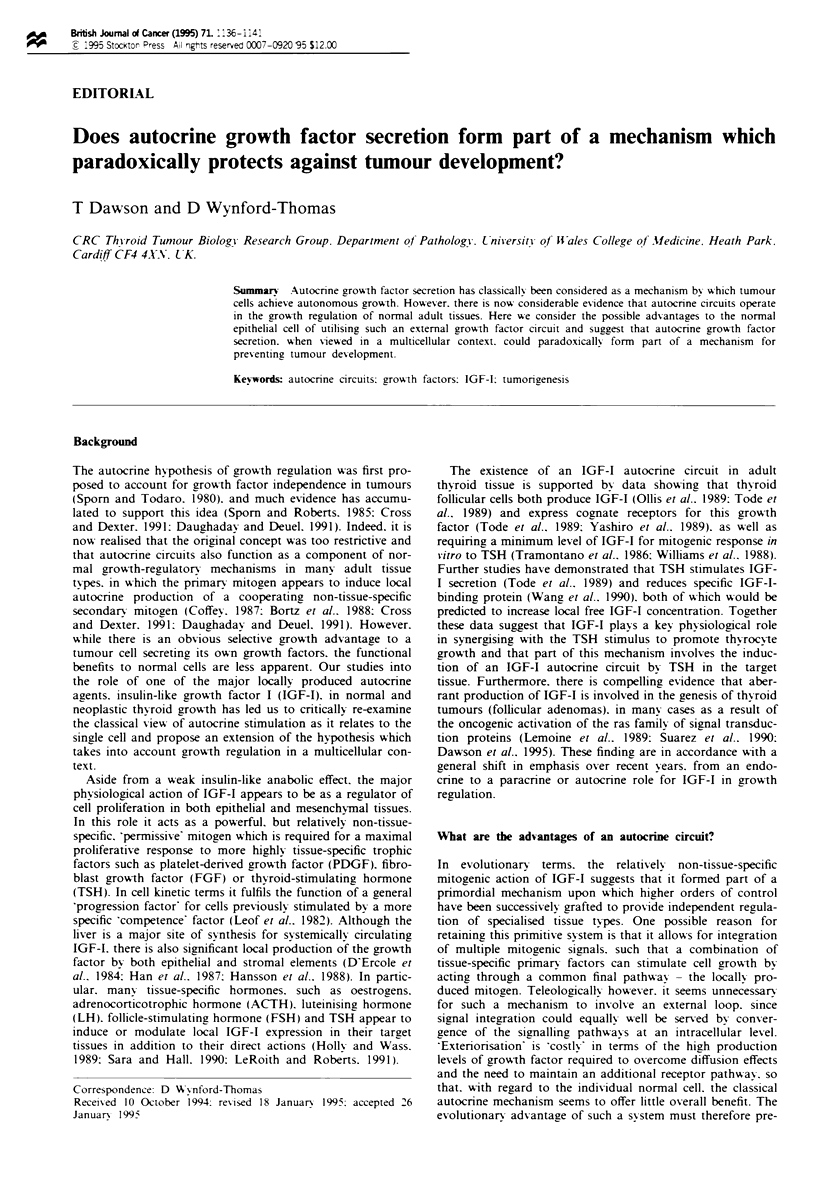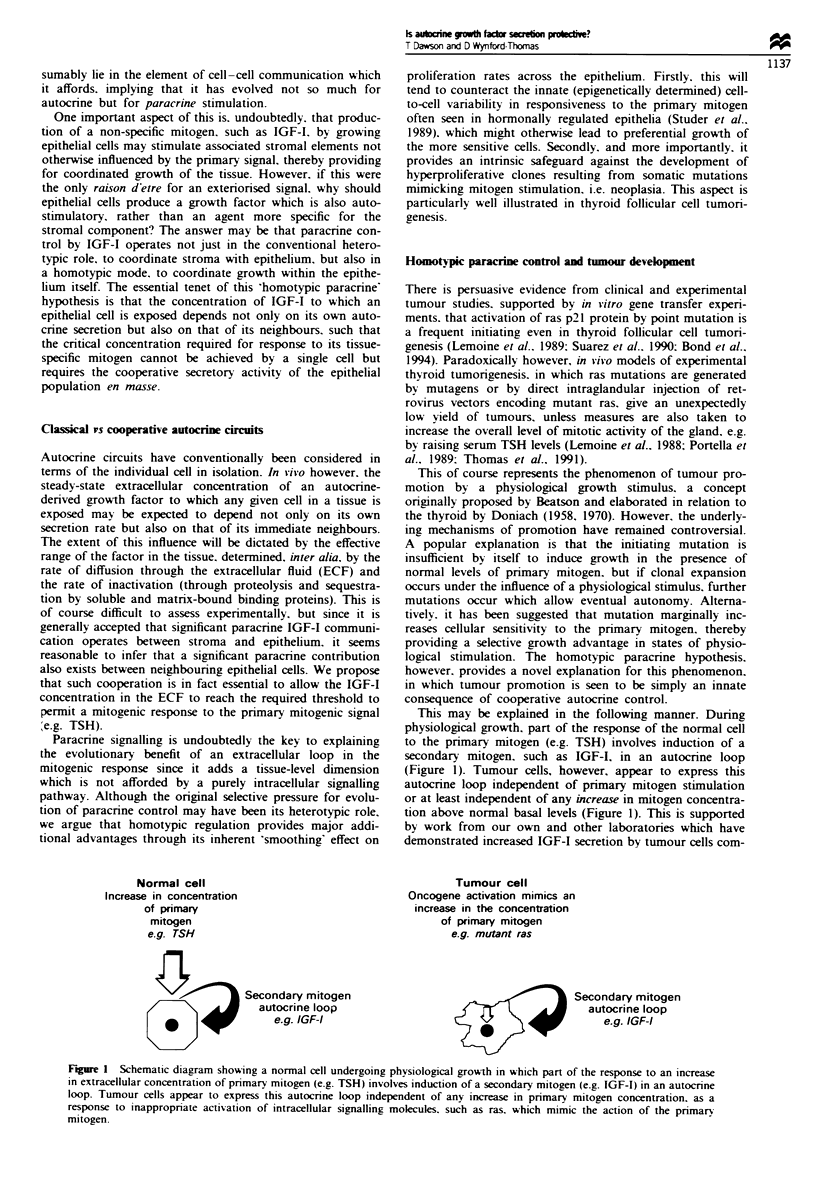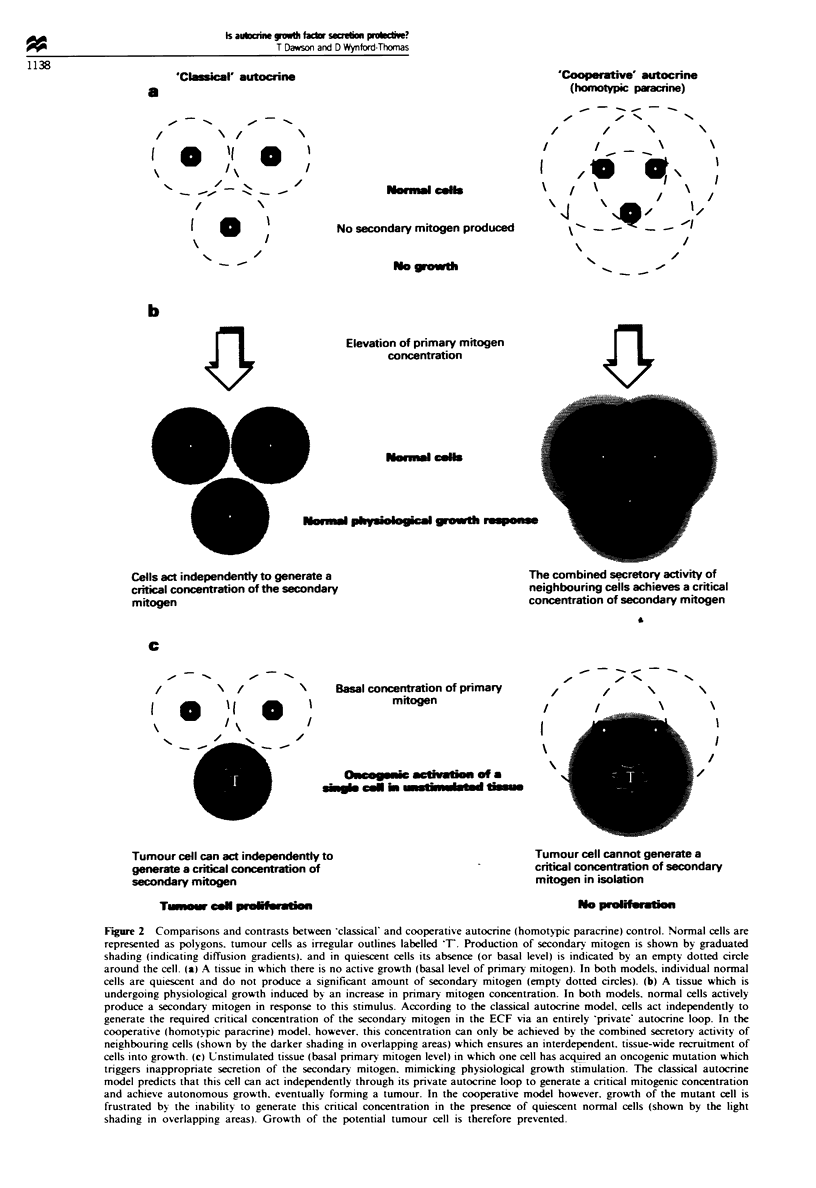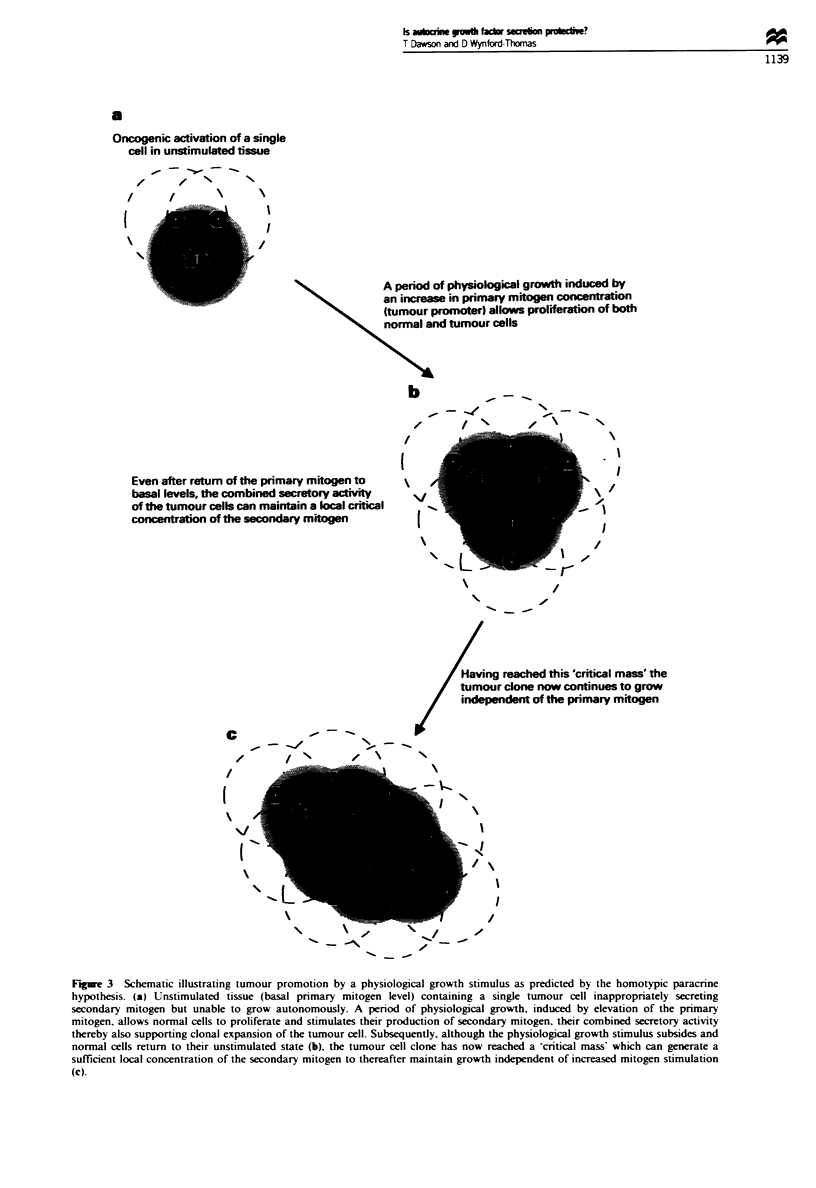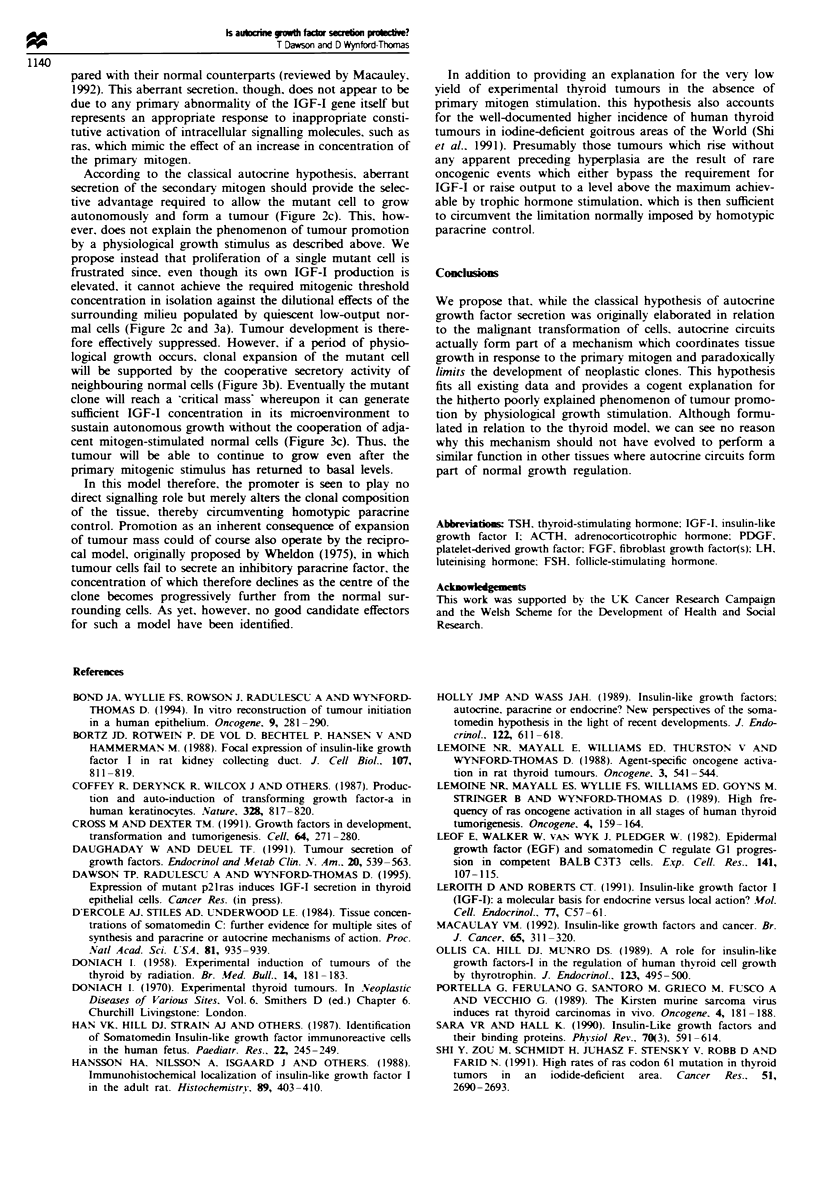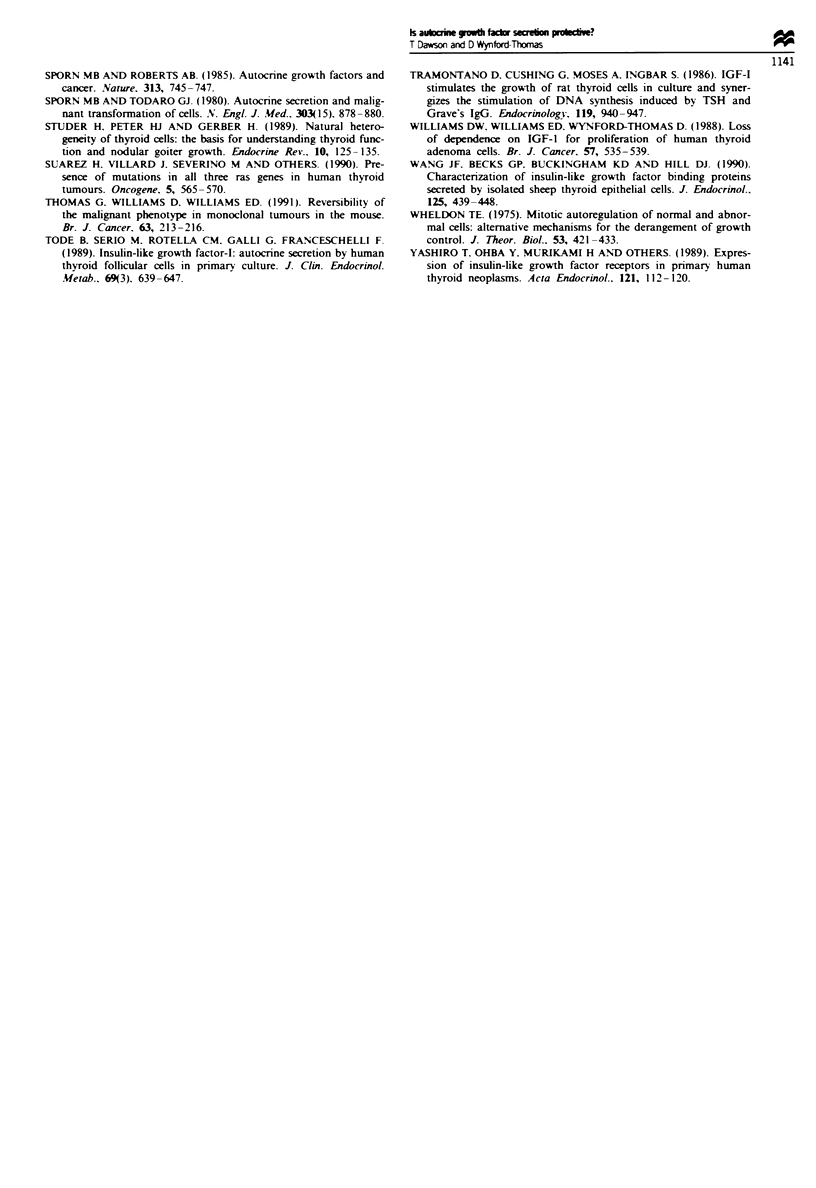# Does autocrine growth factor secretion form part of a mechanism which paradoxically protects against tumour development?

**DOI:** 10.1038/bjc.1995.222

**Published:** 1995-06

**Authors:** T. Dawson, D. Wynford-Thomas

**Affiliations:** Department of Pathology, University of Wales College of Medicine, Heath Park, Cardiff, UK.

## Abstract

Autocrine growth factor secretion has classically been considered as a mechanism by which tumour cells achieve autonomous growth. However, there is now considerable evidence that autocrine circuits operate in the growth regulation of normal adult tissues. Here we consider the possible advantages to the normal epithelial cell of utilising such an external growth factor circuit and suggest that autocrine growth factor secretion, when viewed in a multicellular context, could paradoxically form part of a mechanism for preventing tumour development.


British Journal of Cancer (1995) 71, 1136-1141

© 1995 Stockton Press All rights reserved 0007-0920/95 $12.00

EDITORIAL

### Does autocrine growth factor secretion form part of a mechanism which paradoxically protects against tumour development?

T Dawson and D Wynford-Thomas

*CRC Thyroid Tumour Biology Research Group, Department of Pathology, University of Wales College of Medicine, Heath Park, Cardiff CF4 4XN, UK.*

**Summary** Autocrine growth factor secretion has classically been considered as a mechanism by which tumour cells achieve autonomous growth. However, there is now considerable evidence that autocrine circuits operate in the growth regulation of normal adult tissues. Here we consider the possible advantages to the normal epithelial cell of utilising such an external growth factor circuit and suggest that autocrine growth factor secretion, when viewed in a multicellular context, could paradoxically form part of a mechanism for preventing tumour development.

**Keywords:** autocrine circuits; growth factors; IGF-I; tumorigenesis

### Background

The autocrine hypothesis of growth regulation was first proposed to account for growth factor independence in tumours (Sporn and Todaro, 1980), and much evidence has accumulated to support this idea (Sporn and Roberts, 1985; Cross and Dexter, 1991; Daughaday and Deuel, 1991). Indeed, it is now realised that the original concept was too restrictive and that autocrine circuits also function as a component of normal growth-regulatory mechanisms in many adult tissue types, in which the primary mitogen appears to induce local autocrine production of a cooperating non-tissue-specific secondary mitogen (Coffey, 1987; Bortz *et al.*, 1988; Cross and Dexter, 1991; Daughaday and Deuel, 1991). However, while there is an obvious selective growth advantage to a tumour cell secreting its own growth factors, the functional benefits to normal cells are less apparent. Our studies into the role of one of the major locally produced autocrine agents, insulin-like growth factor I (IGF-I), in normal and neoplastic thyroid growth has led us to critically re-examine the classical view of autocrine stimulation as it relates to the single cell and propose an extension of the hypothesis which takes into account growth regulation in a multicellular context.

Aside from a weak insulin-like anabolic effect, the major physiological action of IGF-I appears to be as a regulator of cell proliferation in both epithelial and mesenchymal tissues. In this role it acts as a powerful, but relatively non-tissue-specific, 'permissive' mitogen which is required for a maximal proliferative response to more highly tissue-specific trophic factors such as platelet-derived growth factor (PDGF), fibroblast growth factor (FGF) or thyroid-stimulating hormone (TSH). In cell kinetic terms it fulfils the function of a general 'progression factor' for cells previously stimulated by a more specific 'competence' factor (Leof *et al.*, 1982). Although the liver is a major site of synthesis for systemically circulating IGF-I, there is also significant local production of the growth factor by both epithelial and stromal elements (D'Ercole *et al.*, 1984; Han *et al.*, 1987; Hansson *et al.*, 1988). In particular, many tissue-specific hormones, such as oestrogens, adrenocorticotrophic hormone (ACTH), luteinising hormone (LH), follicle-stimulating hormone (FSH) and TSH appear to induce or modulate local IGF-I expression in their target tissues in addition to their direct actions (Holly and Wass, 1989; Sara and Hall, 1990; LeRoith and Roberts, 1991).

Correspondence: D Wynford-Thomas
Received 10 October 1994; revised 18 January 1995; accepted 26 January 1995

The existence of an IGF-I autocrine circuit in adult thyroid tissue is supported by data showing that thyroid follicular cells both produce IGF-I (Ollis *et al.*, 1989; Tode *et al.*, 1989) and express cognate receptors for this growth factor (Tode *et al.*, 1989; Yashiro *et al.*, 1989), as well as requiring a minimum level of IGF-I for mitogenic response *in vitro* to TSH (Tramontano *et al.*, 1986; Williams *et al.*, 1988). Further studies have demonstrated that TSH stimulates IGF-I secretion (Tode *et al.*, 1989) and reduces specific IGF-I-binding protein (Wang *et al.*, 1990), both of which would be predicted to increase local free IGF-I concentration. Together these data suggest that IGF-I plays a key physiological role in synergising with the TSH stimulus to promote thyrocyte growth and that part of this mechanism involves the induction of an IGF-I autocrine circuit by TSH in the target tissue. Furthermore, there is compelling evidence that aberrant production of IGF-I is involved in the genesis of thyroid tumours (follicular adenomas), in many cases as a result of the oncogenic activation of the ras family of signal transduction proteins (Lemoine *et al.*, 1989; Suarez *et al.*, 1990; Dawson *et al.*, 1995). These finding are in accordance with a general shift in emphasis over recent years, from an endocrine to a paracrine or autocrine role for IGF-I in growth regulation.

### What are the advantages of an autocrine circuit?

In evolutionary terms, the relatively non-tissue-specific mitogenic action of IGF-I suggests that it formed part of a primordial mechanism upon which higher orders of control have been successively grafted to provide independent regulation of specialised tissue types. One possible reason for retaining this primitive system is that it allows for integration of multiple mitogenic signals, such that a combination of tissue-specific primary factors can stimulate cell growth by acting through a common final pathway – the locally produced mitogen. Teleologically however, it seems unnecessary for such a mechanism to involve an external loop, since signal integration could equally well be served by convergence of the signalling pathways at an intracellular level. 'Exteriorisation' is 'costly' in terms of the high production levels of growth factor required to overcome diffusion effects and the need to maintain an additional receptor pathway, so that, with regard to the individual normal cell, the classical autocrine mechanism seems to offer little overall benefit. The evolutionary advantage of such a system must therefore presumably lie in the element of cell–cell communication which it affords, implying that it has evolved not so much for autocrine but for *paracrine* stimulation.

One important aspect of this is, undoubtedly, that production of a non-specific mitogen, such as IGF-I, by growing epithelial cells may stimulate associated stromal elements not otherwise influenced by the primary signal, thereby providing for coordinated growth of the tissue. However, if this were the only *raison d'etre* for an exteriorised signal, why should epithelial cells produce a growth factor which is also autostimulatory, rather than an agent more specific for the stromal component? The answer may be that paracrine control by IGF-I operates not just in the conventional heterotypic role, to coordinate stroma with epithelium, but also in a homotypic mode, to coordinate growth within the epithelium itself. The essential tenet of this 'homotypic paracrine' hypothesis is that the concentration of IGF-I to which an epithelial cell is exposed depends not only on its own autocrine secretion but also on that of its neighbours, such that the critical concentration required for response to its tissue-specific mitogen cannot be achieved by a single cell but requires the cooperative secretory activity of the epithelial population *en masse*.

### Classical *vs* cooperative autocrine circuits

Autocrine circuits have conventionally been considered in terms of the individual cell in isolation. *In vivo* however, the steady-state extracellular concentration of an autocrine-derived growth factor to which any given cell in a tissue is exposed may be expected to depend not only on its own secretion rate but also on that of its immediate neighbours. The extent of this influence will be dictated by the effective range of the factor in the tissue, determined, *inter alia*, by the rate of diffusion through the extracellular fluid (ECF) and the rate of inactivation (through proteolysis and sequestration by soluble and matrix-bound binding proteins). This is of course difficult to assess experimentally, but since it is generally accepted that significant paracrine IGF-I communication operates between stroma and epithelium, it seems reasonable to infer that a significant paracrine contribution also exists between neighbouring epithelial cells. We propose that such cooperation is in fact essential to allow the IGF-I concentration in the ECF to reach the required threshold to permit a mitogenic response to the primary mitogenic signal (e.g. TSH).

Paracrine signalling is undoubtedly the key to explaining the evolutionary benefit of an extracellular loop in the mitogenic response since it adds a tissue-level dimension which is not afforded by a purely intracellular signalling pathway. Although the original selective pressure for evolution of paracrine control may have been its heterotypic role, we argue that homotypic regulation provides major additional advantages through its inherent 'smoothing' effect on proliferation rates across the epithelium. Firstly, this will tend to counteract the innate (epigenetically determined) cell-to-cell variability in responsiveness to the primary mitogen often seen in hormonally regulated epithelia (Studer *et al.*, 1989), which might otherwise lead to preferential growth of the more sensitive cells. Secondly, and more importantly, it provides an intrinsic safeguard against the development of hyperproliferative clones resulting from somatic mutations mimicking mitogen stimulation, i.e. neoplasia. This aspect is particularly well illustrated in thyroid follicular cell tumorigenesis.

### Homotypic paracrine control and tumour development

There is persuasive evidence from clinical and experimental tumour studies, supported by *in vitro* gene transfer experiments, that activation of ras p21 protein by point mutation is a frequent initiating even in thyroid follicular cell tumorigenesis (Lemoine *et al.*, 1989; Suarez *et al.*, 1990; Bond *et al.*, 1994). Paradoxically however, *in vivo* models of experimental thyroid tumorigenesis, in which ras mutations are generated by mutagens or by direct intraglandular injection of retrovirus vectors encoding mutant ras, give an unexpectedly low yield of tumours, unless measures are also taken to increase the overall level of mitotic activity of the gland, e.g. by raising serum TSH levels (Lemoine *et al.*, 1988; Portella *et al.*, 1989; Thomas *et al.*, 1991).

This of course represents the phenomenon of tumour promotion by a physiological growth stimulus, a concept originally proposed by Beatson and elaborated in relation to the thyroid by Doniach (1958, 1970). However, the underlying mechanisms of promotion have remained controversial. A popular explanation is that the initiating mutation is insufficient by itself to induce growth in the presence of normal levels of primary mitogen, but if clonal expansion occurs under the influence of a physiological stimulus, further mutations occur which allow eventual autonomy. Alternatively, it has been suggested that mutation marginally increases cellular sensitivity to the primary mitogen, thereby providing a selective growth advantage in states of physiological stimulation. The homotypic paracrine hypothesis, however, provides a novel explanation for this phenomenon, in which tumour promotion is seen to be simply an innate consequence of cooperative autocrine control.

This may be explained in the following manner. During physiological growth, part of the response of the normal cell to the primary mitogen (e.g. TSH) involves induction of a secondary mitogen, such as IGF-I, in an autocrine loop (Figure 1). Tumour cells, however, appear to express this autocrine loop independent of primary mitogen stimulation or at least independent of any *increase* in mitogen concentration above normal basal levels (Figure 1). This is supported by work from our own and other laboratories which have demonstrated increased IGF-I secretion by tumour cells com-

**Figure 1** Schematic diagram showing a normal cell undergoing physiological growth in which part of the response to an increase in extracellular concentration of primary mitogen (e.g. TSH) involves induction of a secondary mitogen (e.g. IGF-I) in an autocrine loop. Tumour cells appear to express this autocrine loop independent of any increase in primary mitogen concentration, as a response to inappropriate activation of intracellular signalling molecules, such as ras, which mimic the action of the primary mitogen.

**Figure 2** Comparisons and contrasts between 'classical' and cooperative autocrine (homotypic paracrine) control. Normal cells are represented as polygons, tumour cells as irregular outlines labelled 'T'. Production of secondary mitogen is shown by graduated shading (indicating diffusion gradients), and in quiescent cells its absence (or basal level) is indicated by an empty dotted circle around the cell. (**a**) A tissue in which there is no active growth (basal level of primary mitogen). In both models, individual normal cells are quiescent and do not produce a significant amount of secondary mitogen (empty dotted circles). (**b**) A tissue which is undergoing physiological growth induced by an increase in primary mitogen concentration. In both models, normal cells actively produce a secondary mitogen in response to this stimulus. According to the classical autocrine model, cells act independently to generate the required critical concentration of the secondary mitogen in the ECF via an entirely 'private' autocrine loop. In the cooperative (homotypic paracrine) model, however, this concentration can only be achieved by the combined secretory activity of neighbouring cells (shown by the darker shading in overlapping areas) which ensures an interdependent, tissue-wide recruitment of cells into growth. (**c**) Unstimulated tissue (basal primary mitogen level) in which one cell has acquired an oncogenic mutation which triggers inappropriate secretion of the secondary mitogen, mimicking physiological growth stimulation. The classical autocrine model predicts that this cell can act independently through its private autocrine loop to generate a critical mitogenic concentration and achieve autonomous growth, eventually forming a tumour. In the cooperative model however, growth of the mutant cell is frustrated by the inability to generate this critical concentration in the presence of quiescent normal cells (shown by the light shading in overlapping areas). Growth of the potential tumour cell is therefore prevented.

**Figure 3** Schematic illustrating tumour promotion by a physiological growth stimulus as predicted by the homotypic paracrine hypothesis. (**a**) Unstimulated tissue (basal primary mitogen level) containing a single tumour cell inappropriately secreting secondary mitogen but unable to grow autonomously. A period of physiological growth, induced by elevation of the primary mitogen, allows normal cells to proliferate and stimulates their production of secondary mitogen, their combined secretory activity thereby also supporting clonal expansion of the tumour cell. Subsequently, although the physiological growth stimulus subsides and normal cells return to their unstimulated state (**b**), the tumour cell clone has now reached a 'critical mass' which can generate a sufficient local concentration of the secondary mitogen to thereafter maintain growth independent of increased mitogen stimulation (**c**).

pared with their normal counterparts (reviewed by Macauley, 1992). This aberrant secretion, though, does not appear to be due to any primary abnormality of the IGF-I gene itself but represents an appropriate response to inappropriate constitutive activation of intracellular signalling molecules, such as ras, which mimic the effect of an increase in concentration of the primary mitogen.

According to the classical autocrine hypothesis, aberrant secretion of the secondary mitogen should provide the selective advantage required to allow the mutant cell to grow autonomously and form a tumour (Figure 2c). This, however, does not explain the phenomenon of tumour promotion by a physiological growth stimulus as described above. We propose instead that proliferation of a single mutant cell is frustrated since, even though its own IGF-I production is elevated, it cannot achieve the required mitogenic threshold concentration in isolation against the dilutional effects of the surrounding milieu populated by quiescent low-output normal cells (Figure 2c and 3a). Tumour development is therefore effectively suppressed. However, if a period of physiological growth occurs, clonal expansion of the mutant cell will be supported by the cooperative secretory activity of neighbouring normal cells (Figure 3b). Eventually the mutant clone will reach a 'critical mass' whereupon it can generate sufficient IGF-I concentration in its microenvironment to sustain autonomous growth without the cooperation of adjacent mitogen-stimulated normal cells (Figure 3c). Thus, the tumour will be able to continue to grow even after the primary mitogenic stimulus has returned to basal levels.

In this model therefore, the promoter is seen to play no direct signalling role but merely alters the clonal composition of the tissue, thereby circumventing homotypic paracrine control. Promotion as an inherent consequence of expansion of tumour mass could of course also operate by the reciprocal model, originally proposed by Wheldon (1975), in which tumour cells fail to secrete an inhibitory paracrine factor, the concentration of which therefore declines as the centre of the clone becomes progressively further from the normal surrounding cells. As yet, however, no good candidate effectors for such a model have been identified.

In addition to providing an explanation for the very low yield of experimental thyroid tumours in the absence of primary mitogen stimulation, this hypothesis also accounts for the well-documented higher incidence of human thyroid tumours in iodine-deficient goitrous areas of the World (Shi *et al.*, 1991). Presumably those tumours which rise without any apparent preceding hyperplasia are the result of rare oncogenic events which either bypass the requirement for IGF-I or raise output to a level above the maximum achievable by trophic hormone stimulation, which is then sufficient to circumvent the limitation normally imposed by homotypic paracrine control.

### Conclusions

We propose that, while the classical hypothesis of autocrine growth factor secretion was originally elaborated in relation to the malignant transformation of cells, autocrine circuits actually form part of a mechanism which coordinates tissue growth in response to the primary mitogen and paradoxically *limits* the development of neoplastic clones. This hypothesis fits all existing data and provides a cogent explanation for the hitherto poorly explained phenomenon of tumour promotion by physiological growth stimulation. Although formulated in relation to the thyroid model, we can see no reason why this mechanism should not have evolved to perform a similar function in other tissues where autocrine circuits form part of normal growth regulation.

**Abbreviations:** TSH, thyroid-stimulating hormone; IGF-I, insulin-like growth factor I; ACTH, adrenocorticotrophic hormone; PDGF, platelet-derived growth factor; FGF, fibroblast growth factor(s); LH, luteinising hormone; FSH, follicle-stimulating hormone.

**Acknowledgements**
This work was supported by the UK Cancer Research Campaign and the Welsh Scheme for the Development of Health and Social Research.

### References

BOND JA, WYLLIE FS, ROWSON J, RADULESCU A AND WYNFORD-THOMAS D. (1994). In vitro reconstruction of tumour initiation in a human epithelium. *Oncogene*, **9**, 281-290.

BORTZ JD, ROTWEIN P, DE VOL D, BECHTEL P, HANSEN V AND HAMMERMAN M. (1988). Focal expression of insulin-like growth factor I in rat kidney collecting duct. *J. Cell Biol.*, **107**, 811-819.

COFFEY R, DERYNCK R, WILCOX J AND OTHERS. (1987). Production and auto-induction of transforming growth factor-a in human keratinocytes. *Nature*, **328**, 817-820.

CROSS M AND DEXTER TM. (1991). Growth factors in development, transformation and tumorigenesis. *Cell*, **64**, 271-280.

DAUGHADAY W AND DEUEL TF. (1991). Tumour secretion of growth factors. *Endocrinol and Metab Clin. N. Am.*, **20**, 539-563.

DAWSON TP, RADULESCU A AND WYNFORD-THOMAS D. (1995). Expression of mutant p21ras induces IGF-I secretion in thyroid epithelial cells. *Cancer Res.* (in press).

D'ERCOLE AJ, STILES AD, UNDERWOOD LE. (1984). Tissue concentrations of somatomedin C: further evidence for multiple sites of synthesis and paracrine or autocrine mechanisms of action. *Proc. Natl Acad. Sci. USA*, **81**, 935-939.

DONIACH I. (1958). Experimental induction of tumours of the thyroid by radiation. *Br. Med. Bull.*, **14**, 181-183.

DONIACH I. (1970). Experimental thyroid tumours. In *Neoplastic Diseases of Various Sites*, Vol. 6, Smithers D (ed.) Chapter 6. Churchill Livingstone: London.

HAN VK, HILL DJ, STRAIN AJ AND OTHERS. (1987). Identification of Somatomedin Insulin-like growth factor immunoreactive cells in the human fetus. *Paediatr. Res.*, **22**, 245-249.

HANSSON HA, NILSSON A, ISGAARD J AND OTHERS. (1988). Immunohistochemical localization of insulin-like growth factor I in the adult rat. *Histochemistry*, **89**, 403-410.

HOLLY JMP AND WASS JAH. (1989). Insulin-like growth factors: autocrine, paracrine or endocrine? New perspectives of the somatomedin hypothesis in the light of recent developments. *J. Endocrinol.*, **122**, 611-618.

LEMOINE NR, MAYALL E, WILLIAMS ED, THURSTON V AND WYNFORD-THOMAS D. (1988). Agent-specific oncogene activation in rat thyroid tumours. *Oncogene*, **3**, 541-544.

LEMOINE NR, MAYALL ES, WYLLIE FS, WILLIAMS ED, GOYNS M, STRINGER B AND WYNFORD-THOMAS D. (1989). High frequency of ras oncogene activation in all stages of human thyroid tumorigenesis. *Oncogene*, **4**, 159-164.

LEOF E, WALKER W, VAN WYK J, PLEDGER W. (1982). Epidermal growth factor (EGF) and somatomedin C regulate G1 progression in competent BALB C3T3 cells. *Exp. Cell. Res.*, **141**, 107-115.

LEROITH D AND ROBERTS CT. (1991). Insulin-like growth factor I (IGF-I): a molecular basis for endocrine versus local action? *Mol. Cell. Endocrinol.*, **77**, C57-61.

MACAULAY VM. (1992). Insulin-like growth factors and cancer. *Br. J. Cancer*, **65**, 311-320.

OLLIS CA, HILL DJ, MUNRO DS. (1989). A role for insulin-like growth factors-I in the regulation of human thyroid cell growth by thyrotrophin. *J. Endocrinol.*, **123**, 495-500.

PORTELLA G, FERULANO G, SANTORO M, GRIECO M, FUSCO A AND VECCHIO G. (1989). The Kirsten murine sarcoma virus induces rat thyroid carcinomas in vivo. *Oncogene*, **4**, 181-188.

SARA VR AND HALL K. (1990). Insulin-Like growth factors and their binding proteins. *Physiol Rev.*, **70**(3), 591-614.

SHI Y, ZOU M, SCHMIDT H, JUHASZ F, STENSKY V, ROBB D AND FARID N. (1991). High rates of ras codon 61 mutation in thyroid tumors in an iodide-deficient area. *Cancer Res.*, **51**, 2690-2693.

SPORN MB AND ROBERTS AB. (1985). Autocrine growth factors and cancer. *Nature*, **313**, 745-747.

SPORN MB AND TODARO GJ. (1980). Autocrine secretion and malignant transformation of cells. *N. Engl. J. Med.*, **303**(15), 878-880.

STUDER H, PETER HJ AND GERBER H. (1989). Natural heterogeneity of thyroid cells: the basis for understanding thyroid function and nodular goiter growth. *Endocrine Rev.*, **10**, 125-135.

SUAREZ H, VILLARD J, SEVERINO M AND OTHERS. (1990). Presence of mutations in all three ras genes in human thyroid tumours. *Oncogene*, **5**, 565-570.

THOMAS G, WILLIAMS D, WILLIAMS ED. (1991). Reversibility of the malignant phenotype in monoclonal tumours in the mouse. *Br. J. Cancer*, **63**, 213-216.

TODE B, SERIO M, ROTELLA CM, GALLI G, FRANCESCHELLI F. (1989). Insulin-like growth factor-I: autocrine secretion by human thyroid follicular cells in primary culture. *J. Clin. Endocrinol. Metab.*, **69**(3), 639-647.

TRAMONTANO D, CUSHING G, MOSES A, INGBAR S. (1986). IGF-I stimulates the growth of rat thyroid cells in culture and synergizes the stimulation of DNA synthesis induced by TSH and Grave's IgG. *Endocrinology*, **119**, 940-947.

WILLIAMS DW, WILLIAMS ED, WYNFORD-THOMAS D. (1988). Loss of dependence on IGF-1 for proliferation of human thyroid adenoma cells. *Br. J. Cancer*, **57**, 535-539.

WANG JF, BECKS GP, BUCKINGHAM KD AND HILL DJ. (1990). Characterization of insulin-like growth factor binding proteins secreted by isolated sheep thyroid epithelial cells. *J. Endocrinol.*, **125**, 439-448.

WHELDON TE. (1975). Mitotic autoregulation of normal and abnormal cells: alternative mechanisms for the derangement of growth control. *J. Theor. Biol.*, **53**, 421-433.

YASHIRO T, OHBA Y, MURIKAMI H AND OTHERS. (1989). Expression of insulin-like growth factor receptors in primary human thyroid neoplasms. *Acta Endocrinol.*, **121**, 112-120.